# Supplementary figures and images for: The percentage of CD39+ monocytes is higher in pregnant COVID-19+ patients than in nonpregnant COVID-19+ patients
Source: PLoS One. 2022 Jul 28;17(7):e0264566. doi: 10.1371/journal.pone.0264566 (PMC9333267; doi:10.1371/journal.pone.0264566)

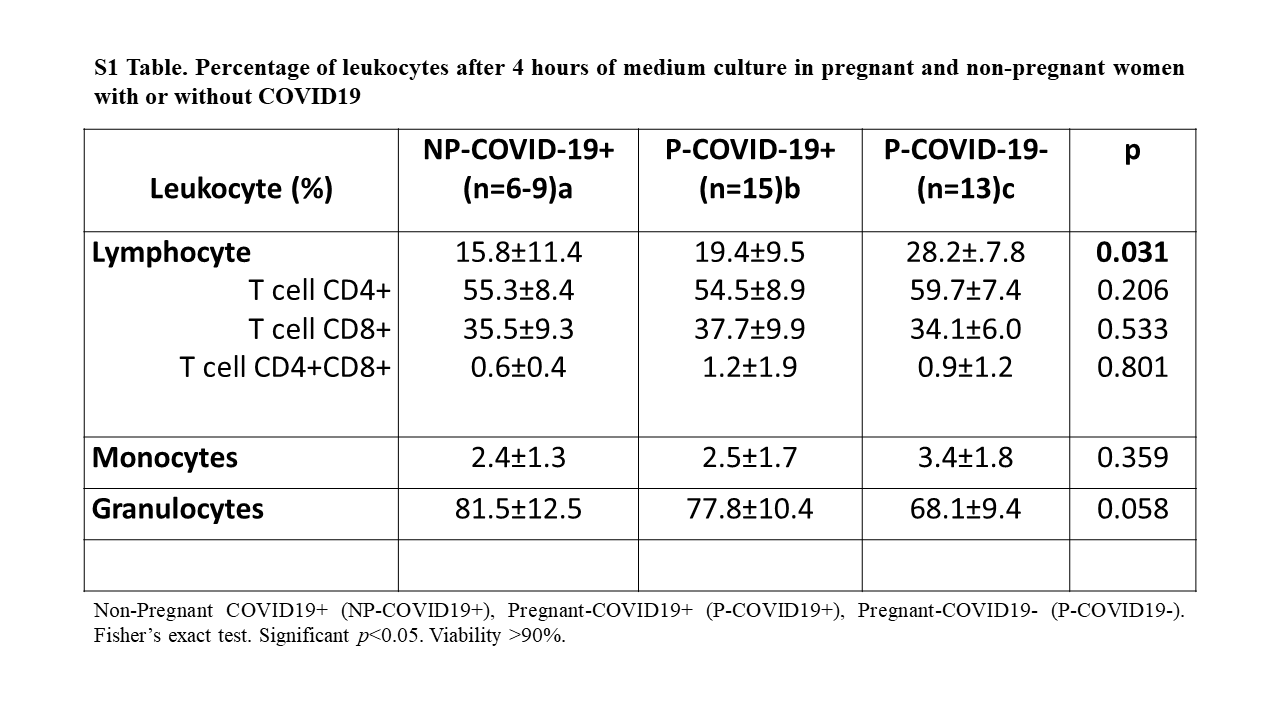

Supplement: S1 Table — (TIF) [file pone.0264566.s001.tif]

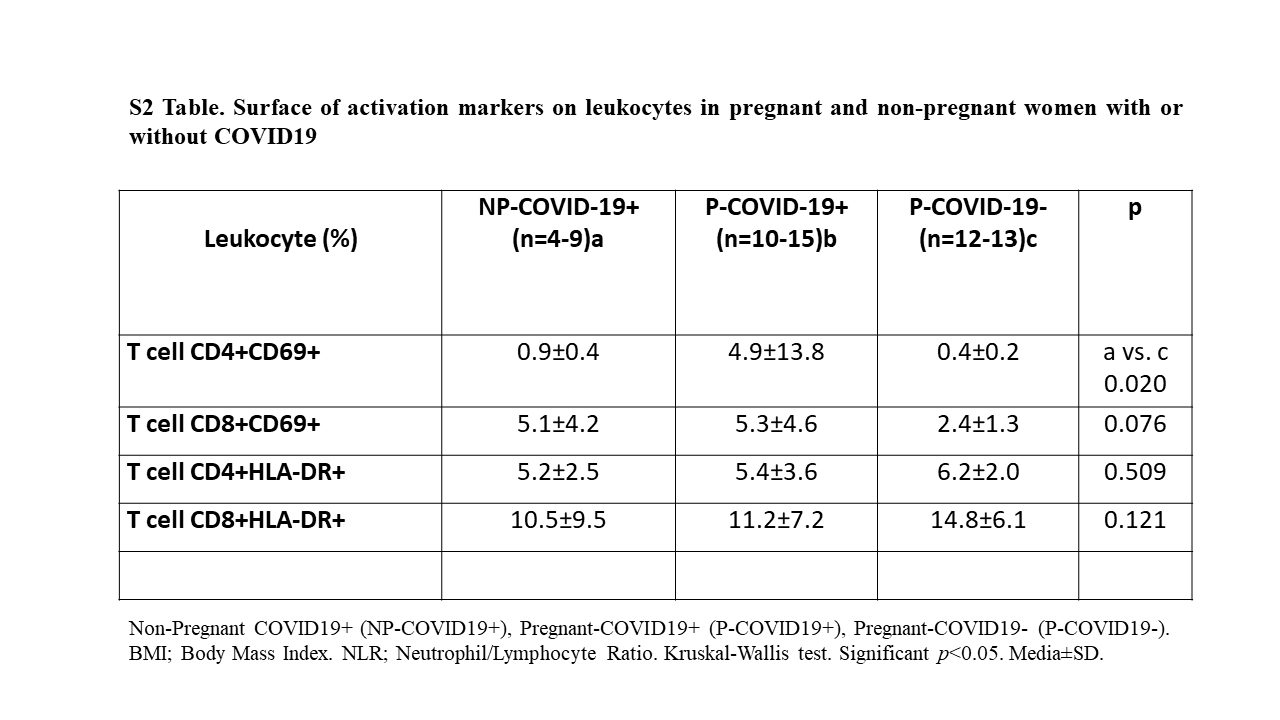

Supplement: S2 Table — (TIF) [file pone.0264566.s002.tif]

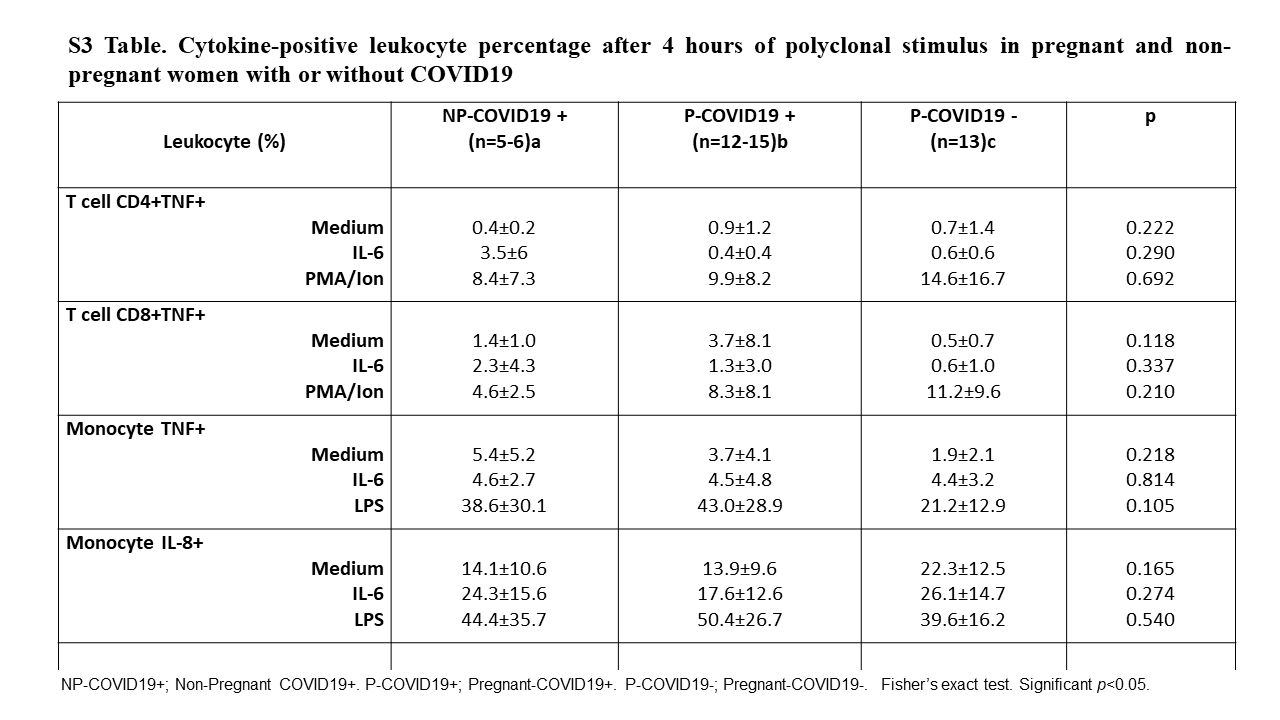

Supplement: S3 Table — (TIF) [file pone.0264566.s003.tif]

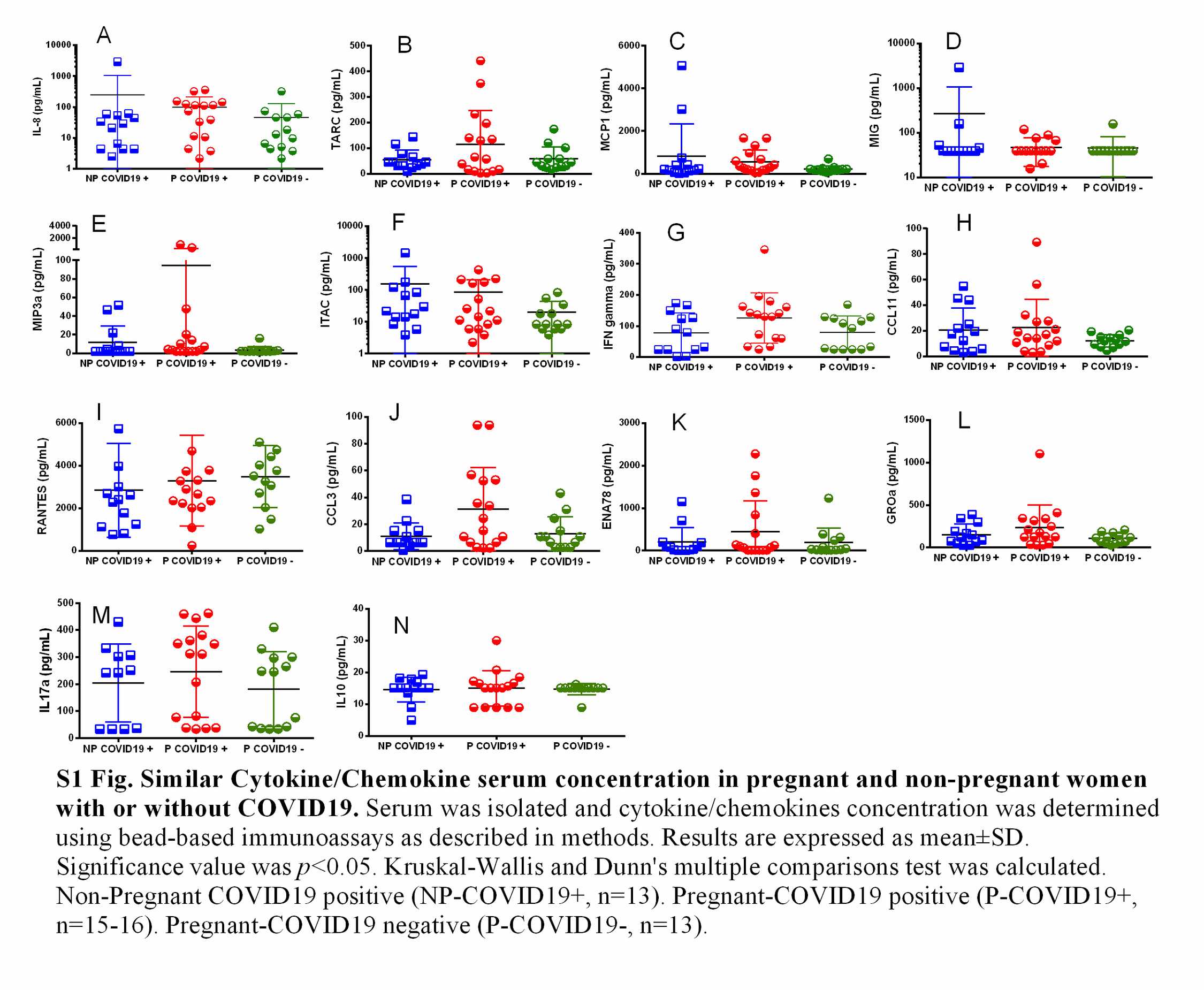

Supplement: S1 Fig — Serum was isolated, and cytokine/chemokine concentrations were determined using bead-based immunoassays as described in the methods. The results are expressed as the mean±SD. Significance value was p<0.05. Kruskal–Wallis and Dunn’s multiple comparisons tests were calculated. Non-Pregnant COVID-19 positive (NP-COVID-19+, n = 13). Pregnant COVID-19 positive (P-COVID-19+, n = 15–16). Pregnant COVID-19 negative (P-COVID-19-, n = 13). (TIF) [file pone.0264566.s004.tif]

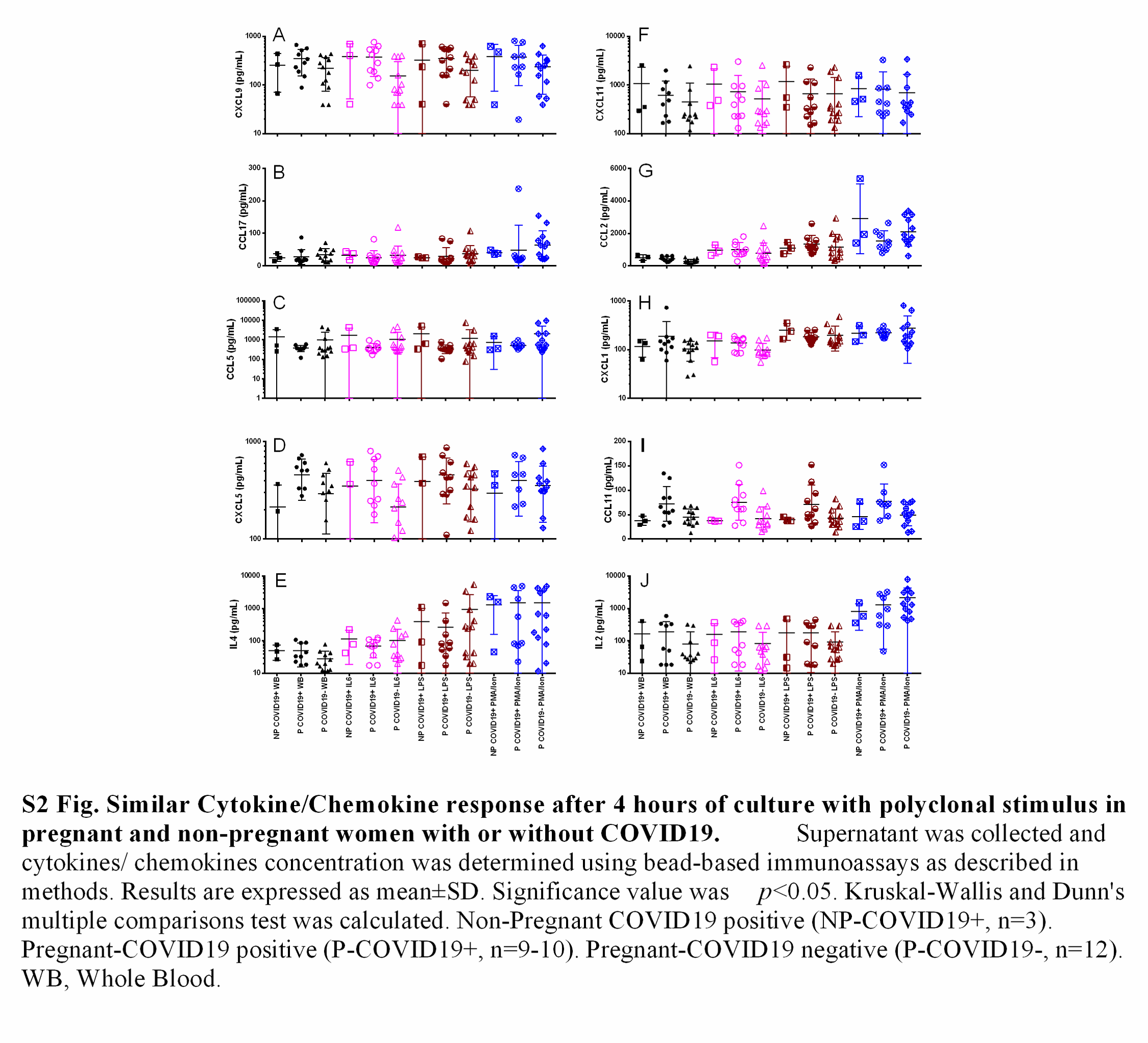

Supplement: S2 Fig — The supernatant was collected, and cytokine/chemokine concentrations were determined using bead-based immunoassays as described in the methods. The results are expressed as the mean±SD. Significance value was p<0.05. Kruskal–Wallis and Dunn’s multiple comparisons tests were calculated. Non-Pregnant COVID-19 positive (NP-COVID-19+, n = 3). Pregnant COVID-19 positive (P-COVID-19+, n = 9–10). Pregnant COVID-19 negative (P-COVID-19-, n = 12). WB, Whole Blood. (TIF) [file pone.0264566.s005.tif]
