# Supplementary material for: The percentage of CD39+ monocytes is higher in pregnant COVID-19+ patients than in nonpregnant COVID-19+ patients
Source: PLoS One. 2022 Jul 28;17(7):e0264566. doi: 10.1371/journal.pone.0264566 (PMC9333267; doi:10.1371/journal.pone.0264566)
Supplement: Attachment — Submitted filename: PLOS ONE Resubmited.docx [file pone.0264566.s007.docx]

Eliseo A Eugenin, Ph.D.

Academic Editor

PLOS ONE

Dear Doctor

Authors want to thank the journal and reviewers, your comments and suggestions really help to improve our manuscript. All points raised by the reviewers were addressed to improve the clarity of the manuscript.

Attending PLOS ONE requirements, we did:

***1. Please ensure that your manuscript meets PLOS ONE's style requirements, including those for file naming.***

**RE**: Now the manuscript meets PLOS ONE's style requirements, including those for file naming.

***2. We note that you have provided funding information within the Acknowledgements Section. Please note that funding information should not appear in the Acknowledgments section or other areas of your manuscript. We will only publish funding information present in the Funding Statement section of the online submission form. Please remove any funding-related text from the manuscript and let us know how you would like to update your Funding Statement.***

**RE:** We remove funding information from the manuscript.

***3. In your Data Availability statement, you have not specified where the minimal data set underlying the results described in your manuscript can be found. PLOS defines a study's minimal data set as the underlying data used to reach the conclusions drawn in the manuscript and any additional data required to replicate the reported study findings in their entirety.***

**RE:** We upload minimal underlying data in an excel file, and verified that all patient´s information is fully anonymized.

***4. Please include captions for your Supporting Information files at the end of your manuscript, and update any in-text citations to match accordingly.***

**RE:** We include captions for supporting information files, and update any in-text citations to match accordingly.

***5. Please amend the manuscript submission data (via Edit Submission) to include author “C. De Lira-Barraza, A. Esquivel-Pineda, G. Flores-Padilla”***

**RE:** We include authors “C. De Lira-Barraza, A. Esquivel-Pineda, and G. Flores-Padilla”

**Comments to the Author**

***6. Conclusions presented are not appropriate and are not supported by the data.
a. The authors did not refer to the purinergic pathway in all sections of the MS, even though the CD39 and CD73 data were available to them.
b. In spite of available data on both CD39 and CD73 authors interpretation of their data and conclusions did not touch on immune suppression/uncontrolled inflammation by [+/-] Adenosine seen in women with a cytokine storm.
c. Several new articles on the subject have not been referred to: (Alahmadi et al, 2020: Cells); (Alberca et al 2020 Frontiers in Immunology); (Chen et al, 2021 Signal transduction); (Dorneles et al, 2021; MedRxiv).***

**RE:** We apologize if the first version of our manuscript expresses conclusions not appropriate and not supported by the data, we modified the text and hope that this mistake have been solved now. **The changes are highlighted in yellow in the manuscript**. In addition, we have cited the articles suggested by the reviewers which now appear in reference section.

***7. The article is presented in very poor English. It must be edited by an English native speaker.***

**RE:** The manuscript English editing (language, grammar, punctuation, spelling, and overall style) was performed by AJE which is one the most prestigious qualified native English-speaking editors. We include the editing certificate we received from them.

***8. The manuscript is very interesting exploring a new immunological field. In my opinion the Authors should better explain the maternal-fetal tolerance that is the key point of the current research. Please mark the suggestive clinical and laboratory characteristics.***

*RE:* We agree that the maternal-fetal tolerance is the key point of the current research, however, functional analysis was limited and we want to be prudent at this respect.

Finally, while performing the manuscript revision to address the reviewers’ comments we identified an error: two patients were wrongly assigned to P-COVID-19+ group; one of them belonged to the NP-COVID-19+ group and the other to the P-COVID19- group. With the corrected groups, we performed the statistical analysis for all demographic, clinical, cytokine/chemokine, immunophenotype and functional data. Despite some p values changed in the significance value (<0.001 vs <0.05, for example), it did not change neither the significance nor the direction of the results. We clarify that the messages of the figures (even with the corrections) are the same, as well as the general conclusion. We apologize for this mistake and, in the understanding that true data is the essence of science, we really appreciate the opportunity to present you this improve and corrected version of our manuscript.

Sincerely yours,

Corresponding author:

Arturo Cérbulo Vázquez. MD. PhD

Hospital General de México “Dr. Eduardo Liceaga”

E-mail: [cerbulo@unam.mx](mailto:cerbulo@unam.mx)
